# Hepatic metastasis complicated by abscess formation

**DOI:** 10.12669/pjms.314.6885

**Published:** 2015

**Authors:** Liao Yi, Qiu Lihua, Diao Xianming, Gong Qiyong

**Affiliations:** 1 Liao Yi, MD. Huaxi MR Research Center (HMRRC), Department of Radiology, West China Hospital of Sichuan University, China 610041. Gong Qiyong, MD, PhD. Huaxi MR Research Center (HMRRC), Department of Radiology, West China Hospital of Sichuan University, China 610041; 2 Qiu Lihua, PhD. Huaxi MR Research Center (HMRRC), Department of Radiology, West China Hospital of Sichuan University, China 610041; 3 Diao Xianming, MS. Department of Radiology, The Second People’s Hospital of Yibin, China 644000; 4 Gong Qiyong, MD, PhD. Huaxi MR Research Center (HMRRC), Department of Radiology, West China Hospital of Sichuan University, China 610041

**Keywords:** Hepatic abscesses, Hepatic metastasis, Magnetic Resonance Imaging, Diffusion Weighted Imaging

## Abstract

Hepatic abscesses and hepatic metastasis are common diseases. However, hepatic abscesses seldom occur in patients with hepatic metastases. We describe a case of a 67-year-old female patient with abdominal pain in the right upper quadrant. Magnetic resonance imaging revealed several lesions, with the largest lesion displaying features of both hepatic pyogenic abscess and liver metastasis. These features included iso- or hypointense signaling on T1WI and T2WI, hyperintense signaling on diffusion weighted imaging of the thick wall, and mixed hyperintense signal in the center on DWI, as well as dramatic and irregular peripheral enhancement was detected on LAVA dynamic contrast scanning.

Aspiration and culture of the largest lesions revealed *Klebsiella pneumoniae* and a pathologic diagnosis of adenocarcinoma. At this point, the patient admitted a history of colon adenocarcinoma 9 years ago treated with hemicolectomy. Therefore, this patient was considered to have a hepatic pyogenic abscesses complicated by hepatic metastasis. The patient began treatment for the responsible pathogens and underwent chemoembolization of the liver lesions. In special cases, we could attempt to pursue a more detailed search for coexistence of microorganism infection and tumor.

## INTRODUCTION

The pyogenic liver abscess is a type of liver abscess caused by bacteria. Pyogenic bacteria can gain access to the liver by direct extension from contiguous organs or through rich dual blood supply provided by the portal vein and hepatic artery. 1 The clinical presentation of liver abscess is insidious, and fever and right upper quadrant pain are the most common complaints. 2 Additionally, the liver is a common site for metastatic disease because of the aforementioned blood supply. The most common primary sites of origin for metastatic lesions to the liver in adults are the gastrointestinal malignancies.

Magnetic resonance imaging (MRI) is a non-invasive way to detect the abnormalities in the liver and has become essential to the diagnoses of both hepatic abscess and hepatic metastasis.^3^ The different signals can provide information about the lesion from different aspects. Diffusion weighted imaging (DWI) is a common MRI sequence whose principles are based upon measurements of the random Brownian motion of water molecules within a voxel of tissue; DWI is particularly useful in detecting the characteristics of infection or tumor.^4^ Other modalities of clinical testing may also help to differentiate the lesions of the liver.

## CASE REPORT

In this case, a 67-year-old patient presented with abdominal pain in the right upper quadrant for more than one day. Physical examination demonstrated right upper quadrant tenderness without rebound or guarding. Carcinoembryonic antigen (CEA) level was 35.34 ng/ml. She did not have past medical history of hepatitis, tuberculosis or malaria.

**Fig. 1:**
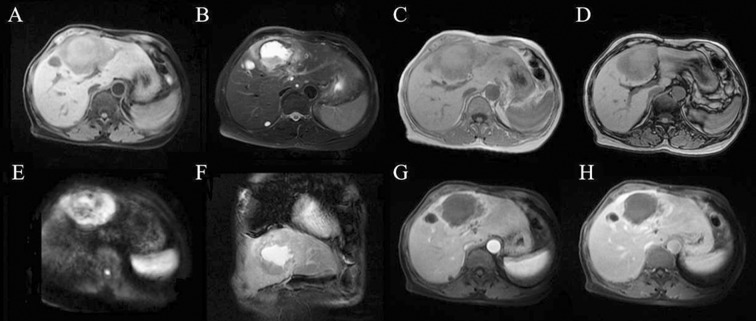
The different sequences of MRI showing the hepatic abscess in the area of the liver metastasis. T1WI (Fig.1 A), axial T2WI (Fig.1 B), coronal T2WI (Fig.1 F), dual echo T1WI (Fig.1 C, D), DWI (Fig.1 E), and LAVA (Fig.1 G, H) were performed.

MR of the abdomen was performed through a 1.5-Tesla MR Scanner (SignaHDx; GE Medical Systems, Milwaukee, WI, USA). Axial T1 Weighted Image (T1WI) (Fig.1A), axial T2 Weighted Image (T2WI) (Fig.1B), coronal T2WI (Fig.1F), dual echo T1WI (Fig.1C, 1D), DWI (Fig.1E), and Liver Acquisition with Volume Acceleration (LAVA) (Fig.1G, 1H) were performed. T1-weighted and T2-weighted MRI demonstrated several abnormal lesions with different signals in the liver. The largest lesion, located in the left lobe of the liver, was irregular in appearance with mixed signals. Specifically, this lesion appeared isointense and moderately hypointense on T1WI, mixed hyperintensity on T2WI in the center as well as hypointense on T1WI, and isointense and moderately hypointense on T2WI in the thick wall. DWI showed a mixed hyperintense signal in the center and a hyperintense signal in the thick wall. A dramatic and irregular peripheral enhancement was detected on Liver Acquisition with Volume Acceleration (LAVA) dynamic contrast scanning. The dual echo T1WI showed no different signals in the mass, indicating no fat was contained in the mass. Several other cystic lesions were demonstrated in the liver with hypointense signal on T1WI, hyperintense signal on T2WI, and a dramatic and progressive ring-like enhancement on LAVA.

The largest lesion was aspirated and culture returned positive for *Klebsiella pneumoniae*; pathological examination further demonstrated adenocarcinoma. The immunohistochemical studies revealed tumor cell positivity for cytokeratin 19, carcinoembryonic antigen (partial positive), villin, caudal-related homeodomain transcription-2, and Ki67 50%, but studies were negative for TTF-1, cytokeratin 7, cytokeratin 20, and NapsinA. Combining the aspirated pus and the pathological results, we established the diagnosis of liver metastasis complicated by hepatic pyogenic abscess. Retrospective clinical history indicated that the patient had a history of colon adenocarcinoma 9 years ago, for which she underwent a hemicolectomy but did not receive chemotherapy.

The patient began treatment for the responsible pathogens and she underwent chemoembolization of the liver lesions, after which she demonstrated clinical improvement.

## DISCUSSION

Hepatic pyogenic abscesses usually arise from portal pyaemia of various causes. The etiologies of these abscesses are divided into six categories based on the extension route of infection: 1) through the bile ducts, 2) by way of the portal vein, 3) by direct extension, 4) from blunt or penetrating trauma, 5) through the hepatic artery, or 6) of obscure origin, or cryptogenic, where no primary source of infection is found, even after abdominal exploration or autopsy.^5^ In all of these cases, an initial local inflammatory is followed by progressive central liquefaction with a surrounding inflammatory margin or “wall”.

Various imaging modalities can help diagnose hepatic pyogenic abscesses. Among those tests, MRI is a non-invasive way to detect the hepatic abscess. The center of a liver abscess often appears as mixed signals with hypointense signaling on T1WI and hyperintense signaling on T2WI. The wall of the abscesses is peripherally enhanced. Additionally, abscesses tend to demonstrate high signaling within the abscess cavity on DWI. This DWI signal is a sensitive marker to detect the hepatic abscesses. Purulent content of an abscess restricts the diffusion of water molecules, resulting in a hyperintense appearance. In this case, the center of the biggest lesions demonstrated the hyperintense signaling on DWI.

A liver metastasis is a malignant tumor in the liver that has spread from another organ affected by cancer. During the development of invasive tumors, tumor cells disobey the social order of organ boundaries and cross into foreign tissues.^6^ Cancers that can spread to the liver include gastrointestinal cancer, breast cancer, lung cancer, and pancreatic cancer. Sometimes the metastatic lesion is identified at the same time as the original cancer; however, sometimes the metastatic lesion is discovered several years after the original cancer has been treated or surgically removed. The appearance of liver metastasis on MRI is variable, but most frequently metastasis appear as moderately hypointense signals on T1WI and moderately hyperintense signals on T2WI. Enhancement may be lesional or perilesional. Additionally, small lesions (< 1.5cm) tend to uniformly enhance while larger lesions (> 1.5cm) most commonly demonstrate transient rim enhancement (i.e. with wash-out). Moreover, perilesional enhancement is most commonly seen in colorectal and pancreatic adenocarcinoma metastasis.

Our patient’s case was rare in that the hepatic metastasis was infected with *Klebsiella pneumoniae*. In view of the widely varying appearances of hepatic abscesses on imaging, and their ability to masquerade as metastatic lesions, it may be necessary to confirm the diagnosis with a needle biopsy.^7^ In this case the liver lesion presented atypically, with characteristics of both liver abscess and liver metastasis. It can be difficult to tell the difference between a liver abscess and a mass due to primary or secondary liver cancer. As demonstrated by this case, these two entities may coexist with characteristics of both the abscess and metastasis. A potential colon malignancy may exist, which should be sought if bacteria are cultured from aspirated pus or blood.^8^,^9^

## References

[1] Choon HT, Tong SK, David JC, Dow MK (2010). Perfusion magnetic resonance imaging of the liver. World J Gastroenterol.

[2] Rubin RH, Swartz MN, Malt R (1974). Hepatic abscess: changes in clinical, bacteriologic and therapeutic aspects. Am J Med.

[3] Zech CJ, Grazioli L, Breuer J, Reiser MF (2008). Diagnostic Performance and Description of Morphological Features of Focal Nodular Hyperplasia in Gd-EOB-DTPA-Enhanced Liver Magnetic Resonance Imaging: Results of a Multicenter Trial. Investigative Radiology.

[4] Taouli B, Koh DM (2010). Diffusion-weighted MR imaging of the liver. Radiology.

[5] Huang CJ, Pitt HA, Lipsett PA, Osterman JFA, Lillemoe KD, Cameron JL (1996). Pyogenic hepatic abscess. Changing trends over 42 years. Ann Surg.

[6] Liotta LA, Steeg PS, Stetler-Stevenson WG (1991). Cancer metastasis and angiogenesis: an imbalance of positive and negative regulation. Cell.

[7] Rockey DC, Caldwell SH, Goodman ZD, Nelson RC, Smith AD (2009). Liver biopsy. American Association for the Study of Liver Diseases. Hepatology.

[8] Teitz S, Guidetti-Sharon A, Hana M, Ariel H (1995). Pyogenic liver abscess: warning indicator of silent colonic cancer. Dis Colon Rectum.

[9] Kahn SP, Lindenauer SM, Wojtalik RS, Hildreth D (1972). Clostridia hepatic abscess: an unusual manifestation of metastatic colon carcinoma. Arch Surg.

